# Stabilization of All Bell States in a Lossy Coupled-Cavity Array

**DOI:** 10.3390/e21040402

**Published:** 2019-04-16

**Authors:** Bing Liu, Dong-Xiao Li, Xiao-Qiang Shao

**Affiliations:** 1 Center for Quantum Sciences and School of Physics, Northeast Normal University, Changchun 130024, China; 2 Center for Advanced Optoelectronic Functional Materials Research, and Key Laboratory for UV Light-Emitting Materials and Technology of Ministry of Education, Northeast Normal University, Changchun 130024, China

**Keywords:** Bell state, quantum-jump-based feedback, dissipative dynamics

## Abstract

A scheme is proposed to generate maximally entangled states of two Λ-type atoms trapped in separate overdamped optical cavities using quantum-jump-based feedback. This proposal can stabilize not only the singlet state, but also the other three triplet states by alternating the detuning parameter and relative phase of the classical fields. Meanwhile it is convenient to manipulate atoms, and much more robust against spontaneous emission of atoms. The parameters related to the potential experiment are analyzed comprehensively and it is confirmed that the quantum feedback technology is a significant tool for entanglement production with a high fidelity.

## 1. Introduction

It is well known that quantum dissipation is a notorious obstacle for various quantum information processing tasks, such as quantum algorithms [1,2], generation of entanglement [3,4], and quantum communication [5,6,7,8,9], which results from the inevitable interaction between the quantum system and the surrounding environment. However, by virtue of the quantum dissipation, more and more triumphs of quantum computation and quantum information have come, such as dissipative preparations of quantum entanglement [10,11,12,13,14,15,16,17], autonomous quantum error correction [18,19,20,21,22,23,24,25], directional quantum state transfer [26,27,28], and so on. These efforts reveal the spectacular promise of quantum dissipation. In particular, Plenio et al. [10] proposed a scheme to generate entangled states with a model consisting of an atom coupled to two distinct leaky, white-noise-field driven optical cavities, and proved the requisite of noise can play a constructive role in certain tasks. Tacchino et al. [16] showed that a bipartite quantum system can be optically pumped into a maximally entangled steady state by exploiting purely incoherent resources, with the largest concurrence reaching the limiting value of 0.5 if one of the two Bell states is coupled to a bath at a negative effective temperature.

On the other hand, the realization of a purely steady entangled state cannot be ensured by the dissipation alone. Therefore, various manners are combined with the disspation to improve the purity of target state such as purification and feedback control [29,30,31,32,33,34,35]. For instance, based on continuous monitoring of quantum jumps, Wang et al. [29] and Mancini [30] showed that the Markovian feedback scheme is able to increase the steady-state entanglement for two two-level atoms coupled to a single Bosonic mode that is driven and heavily damped. Additionally, Carvalho et al. [31] presented a strategy that is insensitive to detection inefficiencies and robust against errors in the control Hamiltonian. Its model consists of a pair of two-level atoms equally, and resonantly, coupled to a single cavity mode in the limit of where cavity decay rate κ is very large. In [32], they also investigated the effects of different control Hamiltonians and detection processes on entanglement production and showed that the quantum-jump-based feedback can protect highly entangled states against decoherence. Nevertheless, in the above schemes, only the singlet state can be prepared as the restriction on the symmetry of the system and the fidelity of the entangled state is significantly reduced by atomic spontaneous emission. Furthermore it is inconvenient to achieve the local feedback control on a single atom since all atoms are trapped into a cavity.

In this work, we present a flexible scheme to generate different Bell states by suitable regulation of relevant parameters, where two Λ-type atoms are respectively trapped in an array of coupled cavities. The photon hopping rate *G* between two cavity provides an additional controllable parameter to realize different Bell states. The atomic spontaneous emission is drastically suppressed by the adiabatic elimination of the excited states. And we will execute a local feedback control to improve the fidelity of the target state as the leakage of photon from the local cavity modes is detected. Ultimately, the system can be stabilized into the target state and needs no requirement of precise control on the time of evolution. We also make a systemic analysis of the relevant parameters in the current experiment and confirm that the quantum-jump-based feedback is reliably useful in stabilization of all kinds of Bell entangled states with high fidelities.

## 2. Effective Master Equation of the Coupled-Cavity Array System

**Figure 1:**
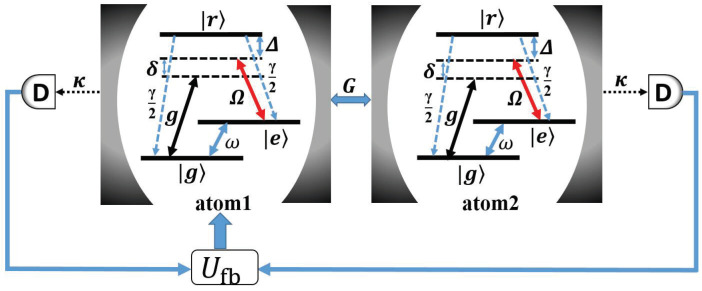
Schematic view of the atom-cavity system. The transition |e〉↔|r〉 is driven by a classical field with a time-independent Rabi frequency Ω; the transition |g〉↔|r〉 is coupled to the cavity with coupling constant *g*; δ and Δ are corresponding detuning parameters. κ is the decay rate of each cavity mode. The transition |g〉↔|e〉 is additionally driven by a microwave field with a Rabi frequency ω and the hopping rate between neighbouring cavities is *G*.

In Figure 1, the system consists of a pair of identical atoms with a Λ-type configuration trapped in a coupled-cavity array and simultaneously driven by classical fields. The transition |g〉↔|r〉 is coupled to a single-mode cavity with a coupling constant *g* and detuning δ+Δ, and the transition |e〉↔|r〉 is driven by a classical field with Rabi frequency Ω and detuning Δ. Additionally, the microwave field is applied to drive the transition |g〉↔|e〉 with Rabi frequency ω. The photon can hop between adjacent cavities with coupling strength *G*. The atoms are assumed to spontaneously radiate from the excited state |r〉 to the ground states |g〉 and |e〉 with an equal decay rate γ/2 and the decay rate of each cavity mode is κ. In a rotating frame, the Hamiltonian of the system reads (ℏ=1).

(1) $$\begin{array}{ccc} H & = & ge^{i\delta t}{(a|r\rangle }_{1}\langle g|+{b|r\rangle }_{2}\langle g|)+\sum_{j=1,2}{\Omega |r\rangle }_{j}\langle e|+\sum_{j=1,2}{\omega |e\rangle }_{j}\langle g|+H.c.\\ & & +\sum_{j=1,2}{\Delta |r\rangle }_{j}\langle r|+G\left(a^{†}b+ab^{†}\right), \end{array}$$

where *a* ($a^{†}$) and *b* ($b^{†}$) denote the annihilation (creation) operators of the local cavities, respectively. In the regime of the large detuning Δ≫{g,Ω}, we can adiabatically eliminate the excited states |r〉 and then the Hamiltonian is simplified as

(2) $$\begin{array}{ccc} H & = & -\frac{g\Omega }{\Delta }e^{i\delta t}{(a|e\rangle }_{1}\langle g|+{b|e\rangle }_{2}\langle g|)+\sum_{j=1,2}{\omega |e\rangle }_{j}\langle g|+H.c.\\ & & -\sum_{j=1,2}\frac{\Omega^{2}}{\Delta }{|e\rangle }_{j}\langle e|-\frac{g^{2}}{\Delta }{(a^{†}a|g\rangle }_{1}\langle g|+b^{†}b{|g\rangle }_{2}\langle g|)+G\left(a^{†}b+ab^{†}\right). \end{array}$$

The Stark-shift terms of ground states |g〉 and |e〉 can be canceled via introducing other ancillary levels, thence the reduced Hamiltonian could be reexpressed as

(3) $$H=-\frac{g\Omega }{\Delta }e^{i\delta t}{(a|e\rangle }_{1}\langle g|+{b|e\rangle }_{2}\langle g|)+\sum_{j=1,2}{\omega |e\rangle }_{j}\langle g|+Ga^{†}b+H.c..$$

In order to achieve a further simplification of the system, we introduce two normal boson models $A=\left(a+b\right)/\sqrt{2}$ and $B=\left(a-b\right)/\sqrt{2}$, which are not coupled to each other but interact with atoms, due to the contributions of the cavity fields. Then we have $a=\left(A+B\right)/\sqrt{2}$ and $b=\left(A-B\right)/\sqrt{2}$ and substitute the relations into Equation (3). The corresponding Hamiltonian is

(4) $$\begin{array}{c} H=-\frac{g\Omega }{\sqrt{2}\Delta }e^{i\delta t}{[A(|e\rangle }_{1}\langle g|+{|e\rangle }_{2}\langle g|)+B{(|e\rangle }_{1}\langle g|-{|e\rangle }_{2}\langle g|)]+\sum_{j=1,2}{\omega |e\rangle }_{j}\langle g|+H.c.+G\left(A^{†}A-B^{†}B\right). \end{array}$$

We further utilize the formula $H_{I}=i{\dot{U}}_{0}^{†}U_{0}+U_{0}^{†}HU_{0}$ to reformulate the Hamiltonian *H* in a rotation with respect to $U_{0}=\exp\left[-itG\left(A^{†}A-B^{†}B\right)\right]$, and

(5) $$\begin{array}{ccc} H_{I} & = & \frac{\tilde{g}}{\sqrt{2}}{[Ae^{i(\delta -G)t}|e\rangle }_{1}\langle g|+Be^{i(\delta +G)t}{|e\rangle }_{1}\langle g|]+\frac{\tilde{g}}{\sqrt{2}}{[Ae^{i(\delta -G)t}|e\rangle }_{2}\langle g|\\ & & -Be^{i(\delta +G)t}{|e\rangle }_{2}\langle g|]+\sum_{j=1,2}{\omega |e\rangle }_{j}\langle g|+H.c., \end{array}$$

where $\tilde{g}=-g\Omega /\Delta$. Under the assumption of δ=G and $\tilde{g}/\sqrt{2}\ll 2G$, the non-local mode *B* is decoupled to the system, and we have

(6) $$\begin{array}{c} H_{\mathrm{eff}}=\sum_{j=1,2}\frac{\tilde{g}}{\sqrt{2}}{(A|e\rangle }_{j}\langle g|+A^{†}{|g\rangle }_{j}\langle e|)+\omega {(|e\rangle }_{j}\langle g|+{|g\rangle }_{j}\langle e|). \end{array}$$

Now we obtain an effective model as in Reference [31], where a pair of two-level atoms are simultaneously driven by a quantum field and a classical field. The difference is that |g〉 and |e〉 are the ground states and mode *A* is non-local. It should be noted that Stevenson et al. have also derived a similar model with two Raman three-level systems in the same cavity [36], but we will see below that the coupled-cavity array system is much useful for stabilizing different Bell states. The dissipative dynamics of the whole system is characterized by the Markovian master equation

(7) $$\begin{array}{ccc} \dot{\rho } & = & -i\left[H_{\mathrm{eff}},\rho \right]+\frac{\kappa }{2}\left(2A\rho A^{†}-A^{†}A\rho -\rho A^{†}A\right), \end{array}$$

where the terms characterizing the spontaneous emission of atom has been neglected, since the atom is restricted in the subspace spanned by {|g〉, |e〉}. By virtue of the standard adiabatic elimination of the non-local cavity mode *A* in the regime of $\kappa \gg \tilde{g}$, the master equation of the reduced density operator for the atomic degrees of freedom becomes (the detail has been shown in the Appendix A)

(8) $$\begin{array}{ccc} \dot{\rho } & = & -i\omega \left[\left(J_{+}+J_{-}\right),\rho \right]+\Gamma D\left[J_{-}\right]\rho , \end{array}$$

where $J_{-}=J_{+}^{†}=\sum_{j=1,2}{|g\rangle }_{j}\langle e|=\sum_{j=1,2}\sigma_{-}^{j}$ represents the cavity-loss induced collective decay operator of atoms with decay rate $\Gamma =2{\tilde{g}}^{2}/\kappa$, and the superoperater D is defined as $D\left[O\right]=\left(2O\rho O^{†}-O^{†}O\rho -\rho O^{†}O\right)/2$.

## 3. Stabilization of Different Bell States via Quantum Feedback Control

Quantum feedback control is a compelling method to make the system evolve to the desired state by suitable regulation of relevant parameters. In our scheme, the loss of cavities equivalent to the collective decay of atoms can be monitored by a photondetector **D**. Once the photons out of cavities are detected by the photondetector, a feedback operator $U_{\mathrm{fb}}=\exp{[-i\lambda (|g\rangle }_{1}\langle e|+{|e\rangle }_{1}\langle g|)]=\exp\left(-i\lambda \sigma_{x}^{1}\right)$ will be applied to the first atom. In this situation the master equation Equation (8) can be modified to [31]

(9) $$\begin{array}{ccc} \dot{\rho } & = & -i\omega \left[\left(J_{+}+J_{-}\right),\rho \right]+\Gamma D\left[U_{\mathrm{fb}}J_{-}\right]\rho . \end{array}$$

In the following numerical simulations, we will characterize the long-time dynamics of system by solving Equation (9) from different initial states instead of direct solving the steady master equation, because there is not always a unique steady solution to Equation (9).

**Figure 2:**
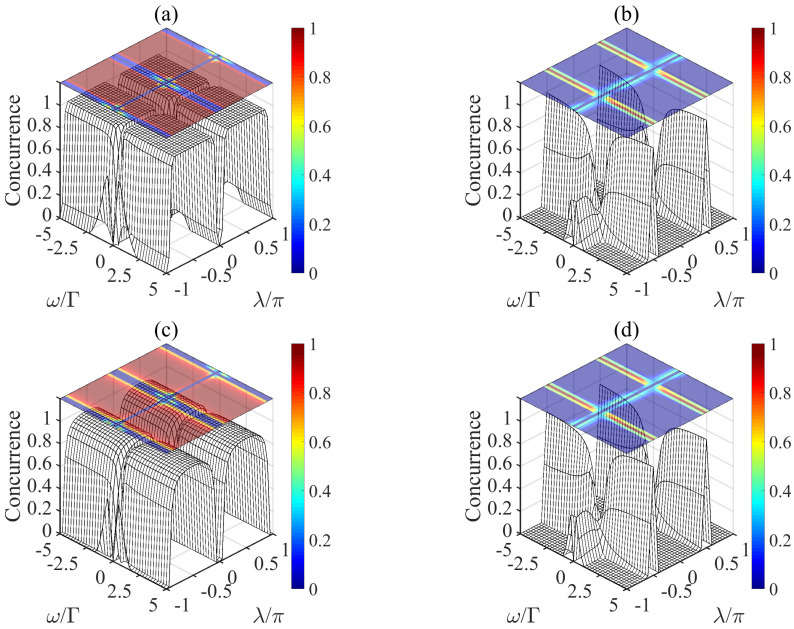
The concurrence of the system at the time Γt=2000 as a function of λ/π and ω/Γ as initial state is $|\phi_{1}{\rangle =|g\rangle }_{1}{|g\rangle }_{2}$, without (**a**,**b**) or with (**c**,**d**) a large decay rate of atomic spontaneous emission (γ=0.1g).

In Figure 2a, we first plot the concurrence of the evolution of the system governed by Equation (9) from an initial state $|\phi_{1}{\rangle =|g\rangle }_{1}{|g\rangle }_{2}$. We find that the concurrence is equal to unity in most of the region except for the point ω/Γ=0 or λ=nπ, and it is also consistent with the result of Reference [31]. A simple inspection shows that the final density matrix ρ is:

(10) $$\begin{array}{ccc} \rho & = & \left(\begin{array}{cccc} 0 & 0 & 0 & 0\\ 0 & 0.5 & -0.5 & 0\\ 0 & -0.5 & 0.5 & 0\\ 0 & 0 & 0 & 0 \end{array}\right), \end{array}$$

which just corresponds to the density matrix of entanglement $|\varphi_{-}\rangle =(|ge\rangle -|eg\rangle )/\sqrt{2}$.

In order to produce other Bell states, we now change the phase of the microwave field acting on the second atom and the corresponding master equation for this case is derived as:

(11) $$\begin{array}{ccc} \dot{\rho } & = & -i\omega \left[\left(\sigma_{x}^{1}-\sigma_{x}^{2}\right),\rho \right]+\Gamma D\left[U_{\mathrm{fb}}J_{-}\right]\rho , \end{array}$$

where $\sigma_{x}^{j}={|g\rangle }_{j}\langle e|+{|e\rangle }_{j}\langle g|$.

Then we plot the concurrence of the system governed by Equation (11) in Figure 2b. The concurrence is almost vanished except for the particular point λ=±0.5π with a large ω/Γ, where the concurrence is near to unity, and the final density matrix ρ is described as:

(12) $$\begin{array}{ccc} \rho & = & \left(\begin{array}{cccc} 0.5 & 0.0248i & -0.0248i & 0.4950\\ -0.0248i & 0.0025 & -0.0025 & -0.0248i\\ 0.0248i & -0.0025 & 0.0025 & 0.0248i\\ 0.4950 & 0.0248i & -0.0248i & 0.4950 \end{array}\right). \end{array}$$

According to Equation (12), we find that the state of system is not pure at this time but it is similar to the density matrix of entanglement $|\psi_{+}\rangle =(|gg\rangle +|ee\rangle )/\sqrt{2}$, where the fidelity of the state $|\psi_{+}\rangle$ is $\sqrt{\langle \psi_{+}|\rho |\psi_{+}\rangle }=0.9926$. Thus the final state can be regarded as $|\psi_{+}\rangle$. This state has not been created using quantum dissipation before and the mechanism of our scheme can be explained in the following. In the absence of the feedback $U_{\mathrm{fb}}$, the steady state of the system is mixed by {|ee〉,|gg〉,|eg〉,|ge〉}. The state |ee〉 may radiate to the state |eg〉 and |ge〉, which will be further transferred to |gg〉. When a feedback operation $U_{\mathrm{fb}}=\exp\left(-i\lambda \sigma_{x}^{1}\right)$ with λ=±0.5π is introduced, the transitions of |eg〉→|gg〉 and |ge〉→|ee〉 will occur, which means that |eg〉 or |ge〉 is unstable in the presence of feedback. Therefore a non-equilibrium mixed state is generated which has a large overlap with the Bell state $|\psi_{+}\rangle$.

Then we consider the effect of atomic spontaneous emission. The formula for the damping operators of the effective atomic spontaneous emission takes the form of

(13) $$\begin{array}{ccc} \;R_{j} & = & \sqrt{\frac{\gamma \Omega^{2}}{2\Delta^{2}}}{|g\rangle }_{j}\langle e|+\sqrt{\frac{\gamma \Omega^{2}}{2\Delta^{2}}}{|e\rangle }_{j}\langle e|, \end{array}$$

and the corresponding master equations reads

(14) $$\begin{array}{ccc} \dot{\rho } & = & -i\omega \left[\left(\sigma_{x}^{1}\pm \sigma_{x}^{2}\right),\rho \right]+\Gamma D\left[U_{\mathrm{fb}}J_{-}\right]\rho +\sum_{j=1,2}D\left[R_{j}\right]\rho \end{array}$$

with different phases (0 and π) of microwave field for the second atom. We then plot the concurrence of $|\varphi_{-}\rangle =(|ge\rangle -|eg\rangle )/\sqrt{2}$ in Figure 2c and $|\psi_{+}\rangle =(|gg\rangle +|ee\rangle )/\sqrt{2}$ in Figure 2d with γ=0.1g. In Figure 2c, the maximum value of concurrence can reach $c_{\max}=0.97$ in the region of |λ/π|∈[0.15,0.85], |ω/Γ|∈(0,5]. Similarly, in Figure 2d, when |λ/π|=0.5, |ω/Γ|∈[3.5,5], the concurrence may achieve at $c_{\max}=0.96$. It adequately demonstrated the robustness of the scheme against the atomic spontaneous emission.

**Figure 3:**
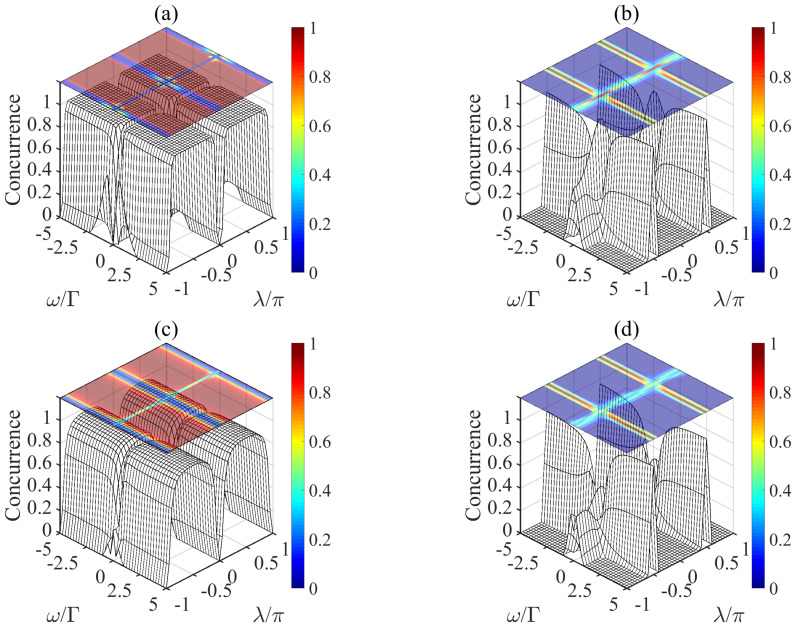
The concurrence of the system in the case of changing the initial state only ($|\phi_{2}{\rangle =|g\rangle }_{1}{|e\rangle }_{2}$) without (**a**,**b**) or with (**c**,**d**) a large decay rate of atomic spontaneous emission (γ=0.1g).

In Figure 3, we consider another initial state $|\phi_{2}\rangle$ as ${|g\rangle }_{1}{|e\rangle }_{2}$, and illustrate the concurrence of the system with atomic spontaneous emission effects (Figure 3c,d) and without atomic spontaneous emission (Figure 3a,b), respectively. For Figure 3a (or Figure 3c), in the region of |λ/π|∈[0.25,0.75], |ω/Γ|∈(0,5], the fidelity of $|\varphi_{-}\rangle =(|ge\rangle -|eg\rangle )/\sqrt{2}$ arrives at $\sqrt{\langle \varphi_{-}|\rho |\varphi_{-}\rangle }=1$ (or $\sqrt{\langle \varphi_{-}|\rho |\varphi_{-}\rangle }=0.97$). Similarly, in Figure 3b (or in Figure 3d), the fidelity of $|\psi_{+}\rangle =(|gg\rangle +|ee\rangle )/\sqrt{2}$ is $\sqrt{\langle \psi_{+}|\rho |\psi_{+}\rangle }=1$ ($\sqrt{\langle \psi_{+}|\rho |\psi_{+}\rangle }=0.96$) for |λ/π|=0.5, |ω/Γ|∈[3.5,5]. Combined with the previous analysis, we can conclude that the preparation of entangled state of our current proposal is independent of the initial state. It is also worth mentioning that in Figure 3b (or Figure 3d), when removing the classical driving field ω/Γ=0 and the feedback coefficient satisfies λ=0.5π, we get the density matrix different from Equation (12):

(15) $$\begin{array}{ccc} \rho & = & \left(\begin{array}{cccc} 0 & 0 & 0 & 0\\ 0 & 0.5 & -0.5 & 0\\ 0 & -0.5 & 0.5 & 0\\ 0 & 0 & 0 & 0 \end{array}\right). \end{array}$$

It means that when the microwave field is absent and we select $|\phi_{2}{\rangle =|g\rangle }_{1}{|e\rangle }_{2}$ and $U_{\mathrm{fb}}=\exp\left(-i\sigma_{x}^{1}\pi /2\right)$, the entangled state $|\varphi_{-}\rangle =(|ge\rangle -|eg\rangle )/\sqrt{2}$ can also be produced. Furthermore, the present scheme can be also used to prepare the states $|\varphi_{+}\rangle =(|ge\rangle +|eg\rangle )/\sqrt{2}$ and $|\psi_{-}\rangle =(|gg\rangle -|ee\rangle )/\sqrt{2}$, which has been discussed in the Appendix B in detail.

**Figure 4:**
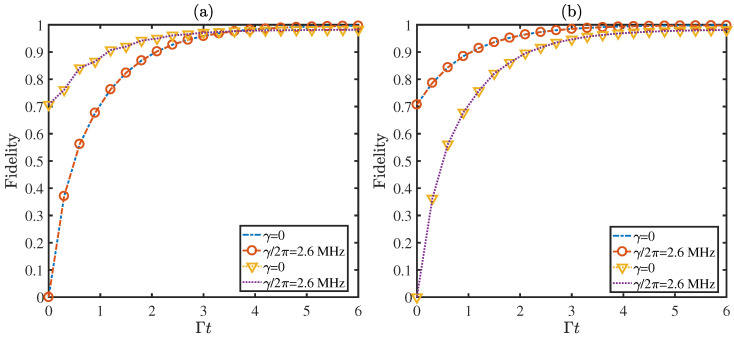
The fidelities for the states $|\varphi_{-}\rangle =(|ge\rangle -|eg\rangle )/\sqrt{2}$ and $|\psi_{+}\rangle =(|gg\rangle +|ee\rangle )/\sqrt{2}$ under different initial states (**a**) $|\phi_{1}{\rangle =|g\rangle }_{1}{|g\rangle }_{2}$, (**b**) $|\phi_{1}{\rangle =|g\rangle }_{1}{|e\rangle }_{2}$ with the full master equation. The selection of related parameters is Ω=g, G=g, Δ=200g, ω/Γ=±5 and the driving frequency is set at λ=0.5π.

Then it is significant to discuss the experimental feasibility of our program. The atomic configuration involved can be realized with Cs atoms. Since the achievement of trapping atoms in cavities has been demonstrated by several groups [37,38,39,40], we can accurately manipulate trapped atoms [37], microwave fields [38] and laser beams [39] to achieve the desired Rabi frequency and atom-cavity coupling. The full master equation of the system is written as

(16) $$\begin{array}{ccc} \dot{\rho } & = & -i\left[H_{I},\rho \right]+\frac{1}{2}\kappa D\left[U_{\mathrm{fb}}a\right]\rho +\frac{1}{2}\kappa D\left[U_{\mathrm{fb}}b\right]\rho +\sum_{j=1,2}D\left[R_{j}\right]\rho , \end{array}$$

where the feedback control is carried out once the photon leaking out of the cavities is detected. In an optical cavity with a coupling coefficient g/2π=10 MHz, the cavity mode and atomic decay rate can be chosen as (κ,γ)/2π=(0.4,2.6) MHz [36,41]. Substituting these parameters and Δ=200g into Equation (16), we acquire the time evolution for fidelities of the target states with different initial states in Figure 4. The purple dashed lines and dotted triangle lines represent the fidelities of the state $|\psi_{+}\rangle =(|gg\rangle +|ee\rangle )/\sqrt{2}$ with and without atomic spontaneous emission, respectively. The other two curves describe the fidelity of state $|\varphi_{-}\rangle =(|ge\rangle -|eg\rangle )/\sqrt{2}$ with (signed empty circles line) and without (blue dashed line) atomic spontaneous emission. Although the atomic dissipation is chosen as γ=2π×2.6 MHz, the fidelities still arrive at 96.01% for $|\psi_{+}\rangle$ and 98.21% for $|\varphi_{-}\rangle$ in a short time Γt=6. It manifests the robustness of our protocol against decoherence once again.

## 4. Summary

In summary, we have put forward an efficient scheme to generate different maximal entanglements by two Λ-type atoms trapped into an array of cavities. After a systematic analysis with the relevant parameters, we confirm the availability of quantum-jump-feedback control to improve the fidelity of entangled states. And our approach is robust against atomic spontaneous emission since the excited states are adiabatically eliminated. In addition, we can obtain all kinds of Bell states by changing the detuning parameter and the relative phase of microwave field operated on two atoms. We hope that our scheme will inspire the future preparation of entangled states with quantum feedback. 

## Appendix A. Derivation of the Effective Master Equation

The dissipative dynamics of the whole system is characterized by the Markovian master equation

(A1) $$\begin{array}{ccc} \dot{\rho } & = & -i\left[H_{\mathrm{eff}},\rho \right]+\frac{\kappa }{2}\left(2A\rho A^{†}-A^{†}A\rho -\rho A^{†}A\right). \end{array}$$

The density operator in the photon number representation is given by [42]

(A2) $$\begin{array}{ccc} \rho & = & \sum_{m,n=0}^{\infty }\rho_{mn}|m\rangle \langle n|, \end{array}$$

where $\rho_{mn}$ are the density matrix elements based on photon number states. When the relation $\kappa \gg \tilde{g}$ is achieved, the highly excited mode of the cavities can be neglected. So the cavity is in the single-photon state at most and the density matrix ρ can be expressed in a small low-photon-number condition to a good approximation, i.e.,

(A3) $$\begin{array}{ccc} \rho & = & \rho_{00}{|0\rangle }_{c}\langle 0|+\rho_{01}{|0\rangle }_{c}\langle 1|+\rho_{10}{|1\rangle }_{c}\langle 0|+\rho_{11}{|1\rangle }_{c}\langle 1|, \end{array}$$

where ${|n\rangle }_{c}$ represents the state of the cavity mode with *n* photons. Substituting Equation (A3) into Equation (A1), we can get a series of coupled equations:

(A4) $$\begin{array}{ccc} {\dot{\rho }}_{00} & = & L\rho_{00}+\frac{i\tilde{g}}{\sqrt{2}}\left(\rho_{01}J_{-}-J_{+}\rho_{10}\right)+\kappa \rho_{11}, \end{array}$$

(A5) $$\begin{array}{ccc} {\dot{\rho }}_{10} & = & L\rho_{10}+\frac{i\tilde{g}}{\sqrt{2}}\left(\rho_{11}J_{-}-J_{-}\rho_{00}\right)-\frac{\kappa }{2}\rho_{10}, \end{array}$$

(A6) $$\begin{array}{ccc} {\dot{\rho }}_{11} & = & L\rho_{11}+\frac{i\tilde{g}}{\sqrt{2}}\left(\rho_{10}J_{+}-J_{-}\rho_{01}\right)-\kappa \rho_{11}. \end{array}$$

The photon-independent items are included in the superoperator $L\rho_{mn}$, where $L\rho_{mn}$ is written as $L\rho_{mn}=i\omega \left[\rho_{mn}\left(J_{+}+J_{-}\right)-\left(J_{+}+J_{-}\right)\rho_{mn}\right]$. In the regime of $\kappa \gg \tilde{g}$, it is reasonable to suppose that $\rho_{10}=0$ and then get the value of the operator as

(A7) $$\begin{array}{ccc} \rho_{10} & = & \rho_{01}^{†}\approx \frac{\sqrt{2}i\tilde{g}}{\kappa }\left(\rho_{11}J_{-}-J_{-}\rho_{00}\right). \end{array}$$

By substituting Equation (A7) into the representation of $\rho_{00}$ and $\rho_{11}$, we can get a diagonal density matrix whose matrix elements are:

(A8) $$\begin{array}{ccc} {\dot{\rho }}_{00} & = & L\rho_{00}-\frac{{\tilde{g}}^{2}}{\kappa }\left(\rho_{00}J_{+}J_{-}-2J_{+}\rho_{11}J_{-}+J_{+}J_{-}\rho_{00}\right)+\kappa \rho_{11}, \end{array}$$

(A9) $$\begin{array}{ccc} {\dot{\rho }}_{11} & = & L\rho_{11}-\frac{{\tilde{g}}^{2}}{\kappa }\left(\rho_{11}J_{-}J_{+}-2J_{-}\rho_{00}J_{+}+J_{-}J_{+}\rho_{11}\right)-\kappa \rho_{11}. \end{array}$$

Because of $\rho_{00}+\rho_{11}=\rho_{\mathrm{atom}}$, these two terms explain the evolutionary nature of the atomic dynamics. We add these two terms together and adiabatically eliminate the term of $\rho_{11}$, the master equation of the simplified density operator for the atomic degrees of freedom becomes:

(A10) $$\begin{array}{ccc} \dot{\rho } & = & -i\omega \left[\left(J_{+}+J_{-}\right),\rho \right]+\Gamma D\left[J_{-}\right]\rho , \end{array}$$

where $\Gamma =2{\tilde{g}}^{2}/\kappa$ denotes the collective decay rate of atoms.

## Appendix B. Generation of the Other Two Triplet States

In order to generate the state $|\varphi_{+}\rangle =(|ge\rangle +|eg\rangle )/\sqrt{2}$, we can choose the δ of Equation (5) as −G, which leads to the non-local mode *B* preserved and *A* dropped. Then we have

(A11) $$\begin{array}{ccc} H_{\mathrm{eff}}^{{}^{\prime }} & = & \sum_{j=1,2}\frac{\tilde{g}}{\sqrt{2}}{[{\left(-1\right)}^{j-1}B|e\rangle }_{j}\langle g|+B^{†}{|g\rangle }_{j}\langle e|]+\omega \left({\sigma_{x}}^{1}\pm {\sigma_{x}}^{2}\right). \end{array}$$

Defining ${\tilde{J}}_{+}={|e\rangle }_{1}\langle g|-{|e\rangle }_{2}\langle g|$, ${\tilde{J}}_{-}={|g\rangle }_{1}\langle e|-{|g\rangle }_{2}\langle e|$, the corresponding master equation of the system becomes

(A12) $$\begin{array}{ccc} \dot{\rho } & = & -i\omega \left[\left({\tilde{J}}_{+}+{\tilde{J}}_{-}\right),\rho \right]+\Gamma D\left[U_{\mathrm{fb}}{\tilde{J}}_{-}\right]\rho , \end{array}$$

and the final density matrix ρ is described as:

(A13) $$\begin{array}{ccc} \rho & = & \left(\begin{array}{cccc} 0 & 0 & 0 & 0\\ 0 & 0.5 & 0.5 & 0\\ 0 & 0.5 & 0.5 & 0\\ 0 & 0 & 0 & 0 \end{array}\right). \end{array}$$

It is equal to the density matrix of the target state $|\varphi_{+}\rangle \langle \varphi_{+}|$ and the preparation of state $|\varphi_{+}\rangle$ is finished.

Similarly, we can set δ=−G of Equation (5) and change the phase of the microwave field for the second atom to generate the state $|\psi_{-}\rangle =(|gg\rangle -|ee\rangle )/\sqrt{2}$. The associated master equation can be rewritten as

(A14) $$\begin{array}{c} \dot{\rho }=-i\omega \left[\left({\tilde{J}}_{+}-{\tilde{J}}_{-}\right),\rho \right]+\Gamma D\left[U_{\mathrm{fb}}{\tilde{J}}_{-}\right]\rho , \end{array}$$

and the final density matrix is

(A15) $$\begin{array}{ccc} \rho & = & \left(\begin{array}{cccc} 0.5 & 0.0248i & 0.0248i & -0.4950\\ -0.0248i & 0.0025 & 0.0025 & 0.0248i\\ -0.0248i & 0.0025 & 0.0025 & 0.0248i\\ -0.4950 & -0.0248i & -0.0248i & 0.4950 \end{array}\right). \end{array}$$

The overlap between this density operator and the ideal state $|\psi_{-}\rangle$ is $F=\sqrt{\langle \psi_{-}|\rho |\psi_{-}\rangle }=0.9926$, which means we have successfully obtained the state $|\psi_{-}\rangle$.
